# Assessing the Strengths and Weaknesses for Implementing a Place-Based Model of Care for Older People on the Central Coast, Australia: Results of a Pilot Project Using the Population Health Management Maturity Index (PHM-MI) Tool

**DOI:** 10.5334/ijic.8575

**Published:** 2024-08-30

**Authors:** Anna Francisca Teresia Maria van Ede, Nicholas Goodwin, Marc Abraham Bruijnzeels, Katharina Viktoria Stein

**Affiliations:** 1 Health Campus The Hague, Leiden University Medical Centre, NL; 2 Central Coast Research Institute for Integrated Care, University of Newcastle and the Central Coast Local Health District, Gosford, New South Wales, AU

**Keywords:** integrated care, joint strategic needs assessment, population health management, Australia, place-based care, older people

## Abstract

**Introduction:**

Population health management is increasingly being used to support place-based models of care. This case study provides an account of the use of the Population Health Management – Maturity Index (PHM-MI) tool to inform the future development of a neighbourhood model of care for older people in the Central Coast region of Australia.

**Description:**

The PHM-MI tool comprises a set of six evidence-informed elements known to be important in enabling PHM in practice. As part of a joint strategic needs assessment, 17 selected stakeholders from key regional organizations were invited to undertake the PHM-MI tool survey. Three follow-up workshops were held to interpret the results and determine priority actions.

**Discussion:**

The PHM-MI scores revealed that the overall maturity of the Central Coast to successfully deliver PHM was low across all six elements, findings that were corroborated through participant workshops. Systemic fragmentations, most pertinently of funding and regulation, incentivised silo-based working. The need to formalise and strengthen regional collaborations, enable data integration, find creative ways to use existing funding streams, and promote community engagement were highlighted as core priorities.

**Conclusion:**

Using the PHM-MI tool was enabled by it being embedded within a pre-existing regional strategic process. The results were used to inform future regional priorities. The PHM-MI tool has the potential for use across regional or national contexts.

## Introduction

The emerging landscape of integrated care demands innovative approaches that transcend traditional boundaries, prioritize the needs of the population, and foster collaboration among key stakeholders, including the people themselves. As emphasized by Stein et.al., the collective sentiment from the past decade of learning about integrated care has been to better understand the question “how to implement integrated care in practice?” [1]. Case examples demonstrate that integrated care programs often struggle to develop or sustain collaboration across different organisational stakeholders working at different levels of the system, resulting in a negative impact on implementation and effectiveness [2]. A key question emerging from this is how integrated care programs can enable key stakeholders to work across their boundaries to collectively improve the health and wellbeing of the populations they serve?

One of the perspectives used to provide a solution to this question is population health management (PHM). PHM is an approach that aims to improve the health and wellbeing of a particular population by developing and implementing interventions based on data analysis and co-creation [3,4]. PHM is a strategy that may help deliver integrated care more effectively by enabling those designing and implementing integrated care to understand the context in which they are seeking to develop their solutions. In this way, it provides an opportunity for stakeholders to come together and develop a more collective response to significant issues in their local health and care system, starting with a total population perspective. Indeed, in recent years, the movement towards integrated care in many countries has begun to take a more ‘place-based’ perspective [5,6,7].

PHM is developed as an approach that in its most narrow form selects the most impactful interventions for the population at risk [4]. Following the definition of the World Health Organisation, it is used as a data driven, proactive managerial approach to look at the whole regional population emphasizing differences within the population and the wider determinants to both select interventions and also implement and evaluate them successfully [8]. However, existing tools that may guide initiatives to implement such a comprehensive PHM approach either have a narrow focus or lack transparency [9]. Therefore, the PHM Maturity Index (PHM-MI) was developed to support stakeholders in assessing the use and progress of PHM elements in their region. Maturity models in other contexts have supported implementation in different ways [10]. For example, the SCIROCCO tool has supported several organizations in European regions to understand the local conditions for integrated care [11].

This case study examines the use of the PHM-MI within an ongoing effort in the Central Coast region of Australia to develop a place-based model of care. Therefore, it focuses on the capability of the Central Coast to lead and deliver such a place-based model of care. Specifically, the paper examines the extent to which the Central Coast is ‘ready for change’ by using the PHM-MI tool to assess the strengths and weaknesses of the region for implementing its place-based model.

## Case description

**Figure 1:**
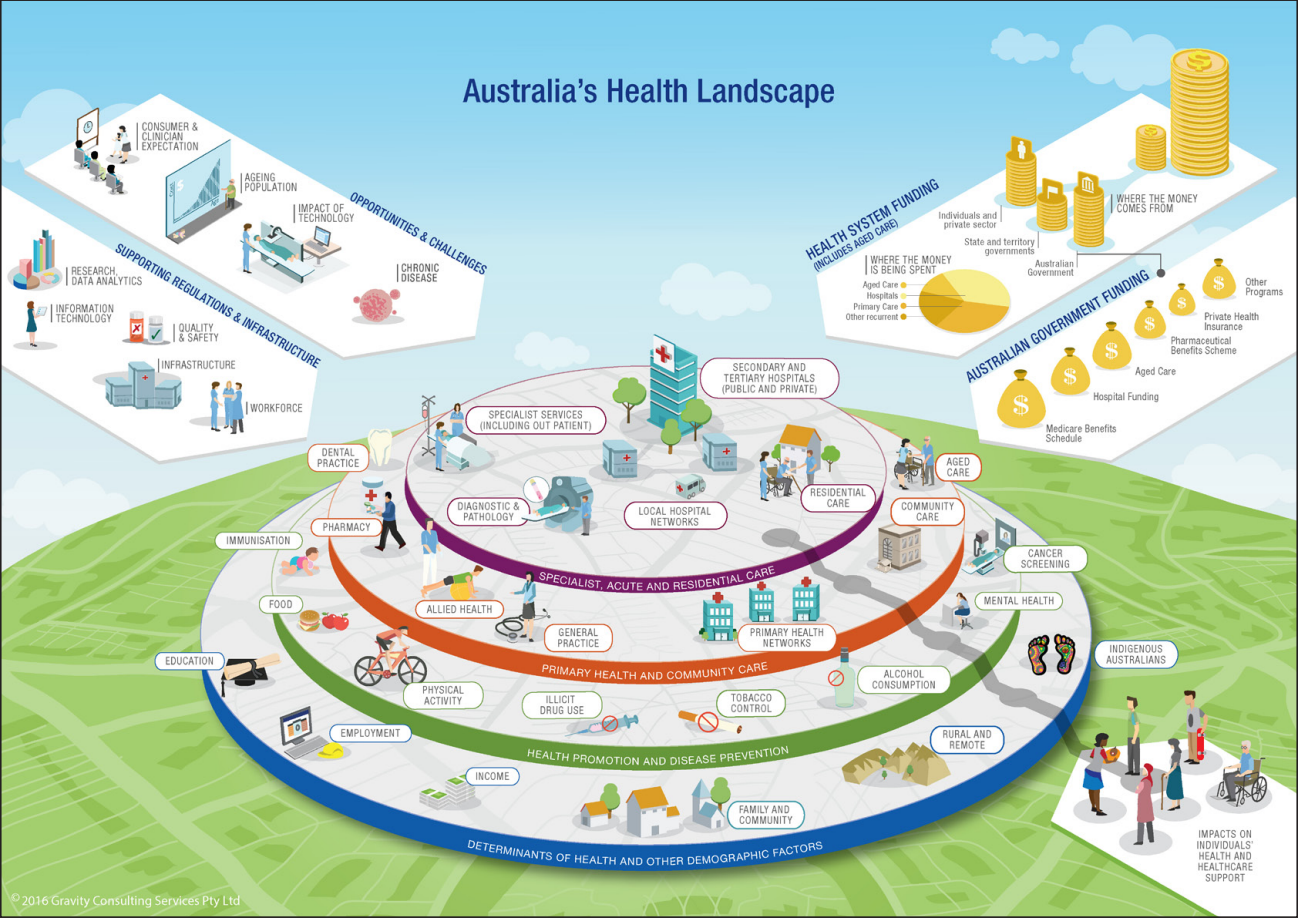
The Australian Health and Care System [14].

The Central Coast is a region on the east coast of Australia, in the state New South Wales (NSW), and covers approximately 350,000 people. The responsibility for the health and wellbeing of Australian community members on the Central Coast is divided between the community members themselves, the private health market, and government agencies. As Figure 1 demonstrates, the funding and delivery landscape is a complex one. At State government level, NSW Health is responsible for funding hospital, community and ambulance services including health promotion and public health services. Most of this responsibility is delegated to the Central Coast Local Health District (CCLHD), who runs two hospitals, two sub-acute facilities and eight community health centres and additional services across the Central Coast region [12]. At Federal government level, funding supports a network of largely privately-run primary and aged care providers, including aged care services in the home. Next to that, the Hunter New England Central Coast Primary Health Network (HNECCPHN), an agency funded by the federal government, strives for improving the efficiency and effectiveness of the primary health care system by managing a range of service agreements with local (private) health providers [13]. The CCLHD and the HNECCPHN work together on shared priorities for best outcomes to people living on the Coast through the Central Coast Health Alliance (the Alliance).

### Developing a place-based model of care on the Central Coast

In September 2022, the Alliance came together with other regional stakeholders, most notably the Central Coast Council, to develop a place-based model of care called *All-Inclusive Care for Older People* (ALICE). The main reason was a shared sense of urgency in developing strategies to meet the impact of changing demographics and service utilisation patterns [15]. Specifically, predictions show that the Central Coast has the fastest ageing population in NSW. In 2020/21, people aged over 70 on the Central Coast represented 16% of the population (some 55,000 people). Yet this subpopulation represented 56% of acute bed stays, 64% of long-stay admissions and over two-thirds of overall costs of care to the health and care system. By 2036, a predicted rise on the Central Coast of an additional 20,000 people aged over 70 (a 39% growth compared to just a 7% growth for the rest of the population) is leading to the following observable systemic pressures:

- Care for older people with complex chronic conditions relies excessively on the hospital systems and curative model of care.
- There is a crowded and fragmented delivery landscape for in-home and community-based services for older people at risk of hospitalisation.
- The complexity of the care system discourages older people from accessing services in primary and community care settings.
- Few services address the wider needs of older people, such as reducing social isolation or using connectedness to community as a means of improving health outcomes.

**Figure 2:**
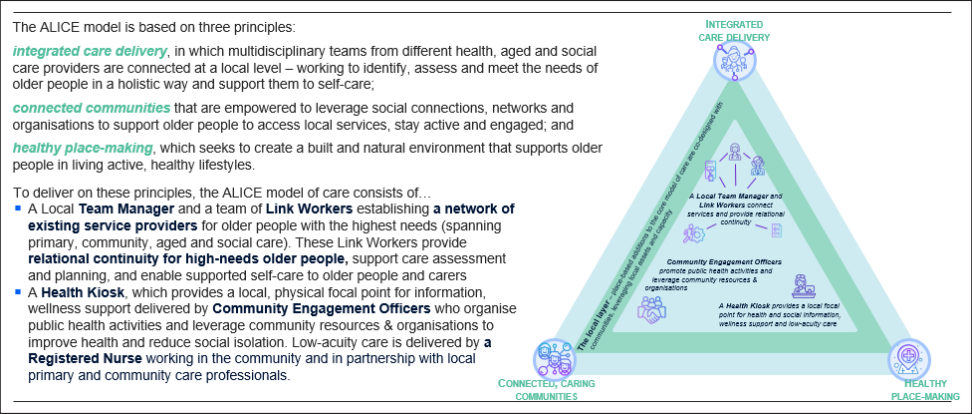
All Inclusive Care for Older People (ALICE): The Emerging Neighbourhood Model of Care on the Central Coast.

The future impact of this growing ageing population has led to an urgent case for change to create a more sustainable approach to provide care and support for the older population in the region. In jointly identifying these needs, the Alliance developed a shared ambition to establish more neighbourhood-based approaches to support older people’s health and wellbeing. This has led to a co-creation activity with the local community to develop the ALICE concept and model of care (see Figure 2).

As part of this shared ambition, the Alliance recognized the need to develop a better understanding of the population health needs on the Central Coast and its localities. In May 2022, a joint strategic needs assessment (JSNA) was commissioned to create a dataset at a locality level to examine these priority needs and feed this information into clinical service planning as well as to identify local communities that may most benefit from place-based care [16]. As part of the JSNA process, the research on which this paper is based was established to engage with key stakeholders at an organizational level to examine strengths and weaknesses of their capability to collectively design and deliver a new model of care for older people in a specific neighbourhood.

## Project description

Exempt for Human Research Ethics Committee (HREC) approval was granted by the CCLHD Research Office (0822–073C). The roles of the authors in relation to the developments on the Central Coast and the PHM-MI were as follows: AE was involved in the initiation, development and writing of the PHM-MI and performed the piloting process on the Central Coast, MB and VS were involved in the initiation and development of the PHM-MI, and NG was project manager of the JSNA, led the development of the place-based initiative (ALICE), and embedded the piloting of the PHM-MI in the ongoing strategic processes on the Central Coast. The full PHM-MI as used in this integrated care case can be obtained from the first author upon request.

## Tool development of the PHM-MI

The PHM-MI is meant to function as a guide to facilitate progress on policy and strategy in a regional setting. It navigates the challenging terrain of building interorganizational collaboration, enabling sustainable change that is responsive to the evolving needs of the population. The tool’s novelty lies in its ability to show stakeholders where the possibilities and challenges lie in their region, which prerequisites they can focus on to better enable care and support that fits population need. By creating an overview of opportunities for a regional strategy, the PHM-MI empowers key stakeholders to build towards a more responsive and adaptable system together and embracing a population-focused approach.

The PHM-MI was developed as an answer to the demand for practice-oriented guidance on where to start and what is needed for sustainable change towards improving the health and wellbeing of the population. The tool seeks to assist regions like the Central Coast by examining current strengths and weaknesses across six key domains known to be supportive to the implementation of a PHM approach. The PHM-MI mainly focuses on supportive conditions that will increase the possibility of success, rather than which exact health or care interventions will be needed in the region.

In the first phase of the development process the content of the tool was constructed by performing a literature review and an expert opinion process (Delphi study) on the items that influence the implementation of PHM [2]. During the first rounds of the expert opinion process, Dutch experts reflected on the items. In the last rounds, an international panel mainly consisting of European researchers validated the included items [9].

The PHM-MI consists of 106 items divided into six elements of PHM:

- Accountable regional organization: This element of the PHM-MI sets out the need for an ‘accountable regional organisation’ that takes collective responsibility for the Quadruple Aim. Often, this is a group of stakeholders that takes the form of a legal entity or alliance, often with specific financial arrangements with payers of health and social care and support.
- Cross-domain business model: The ‘cross domain business model’ is an element of the PHM-MI related to the business of different organisations that come together for the purpose of PHM – specifically, that financial streams are more aligned and the consequences for all regional stakeholders are transparent.
- Integrated data infrastructure: ‘Integrated data infrastructure’ refers to an element of PHM-MI in which routinely registered data in health and social care and support is connected in a sustainable infrastructure to provide a regional comprehensive overview of health, costs and experiences.
- Co-designing workforce and community: ‘Co-designing workforce and community’ represents an element of the PHM-MI that refers to effective structures to co-design initiatives with citizens and local health and care professionals, so ensuring direct participation and a substantial role in the final decision-making process.
- Population health data analytics: ‘Population health data analytics’ is an element of the PHM-MI describing the use of data-driven insights to drive PHM interventions and monitor the Quadruple Aim outcomes regularly. Such data analytics make use of the integrated data infrastructure to provide regional insights.
- Emergent implementation strategies: ‘Emergent implementation strategies’ is an element of the PHM-MI that indicate the presence of a continuous process of testing and learning in the region.

Overall, the tool is designed as a useful framework for organizations to assess their current level of maturity in managing population health together in their region and identify areas for improvement. By focusing on all six elements, organizations can develop a more comprehensive and effective approach that improves outcomes for the populations they serve. In this project, not only was the PHM-MI applied in a real-world setting for the first time, but it was also applied in a different national context than where it was originally developed.

## Implementation process on the Central Coast

To put the PHM-MI into practice, a diverse group of stakeholders on the Central Coast who were already engaged in the JSNA process were invited to join the project. Participants were asked to score the PHM-MI online and then discuss the scores and implications for the Central Coast in three consecutive workshops. 17 stakeholders from the CCLHD, HNECCPHN, Regional NSW, Central Coast Council, Greater Cities Commission and a general practitioner were invited to participate in the project with the possibility to nominate a delegate to join. This number of participants was chosen purposefully to ensure multiple perspectives from those actively engaged in strategic planning and to enable all participants the opportunity to share their point of view in the discussion. A total of 13 participants contributed to the project. Nine of them completed the online survey, four participants attended the first workshop, seven attended the second workshop, and four attended the third workshop. None of the participants filled out the survey and attended all workshops.

Response data were collected and managed using REDCap electronic data capture tools hosted at the CCLHD [17,18]. The PHMI-MI was converted into REDCap, in which additional questions were added to learn about the interpretation of the tool. Afterwards three workshops were held in a timespan of two months to discuss the tool and the results. The first workshop was focused on the interpretation of the tool itself. The second workshop focused on the outcome of the PHM-MI. Guided by the results, the participants were encouraged to interpret the outcome and discuss if this provided an adequate overview of the strengths and weaknesses of the Central Coast. In the third and final workshop the participants discussed the possible next steps to promote integrated care on the Central Coast.

The results of the PHM-MI were analysed using Excel. For each item, the median, agreement score, and highest and lowest scores were calculated. The median score was chosen to diminish the influence of outliers. To calculate the agreement score, the median score was divided into three categories: 1–3 low, 4–6 middle, and 7–9 high. If >70% of the experts scored in the same category as the median, agreement was reached. The difference between the highest and lowest scores showed the degree in variability. Comments from the survey and the results from the workshops were analysed based on the six elements of PHM to verify the meaning of the scores and expand the analysis on strengths and weaknesses of the Central Coast.

## Results

The results section is split into the quantitative data of the scores of the PHM-MI and the qualitative data of the comments from the survey and the workshop data. The qualitative data is displayed per PHM-element.

### Quantitative results

**Figure 3:**
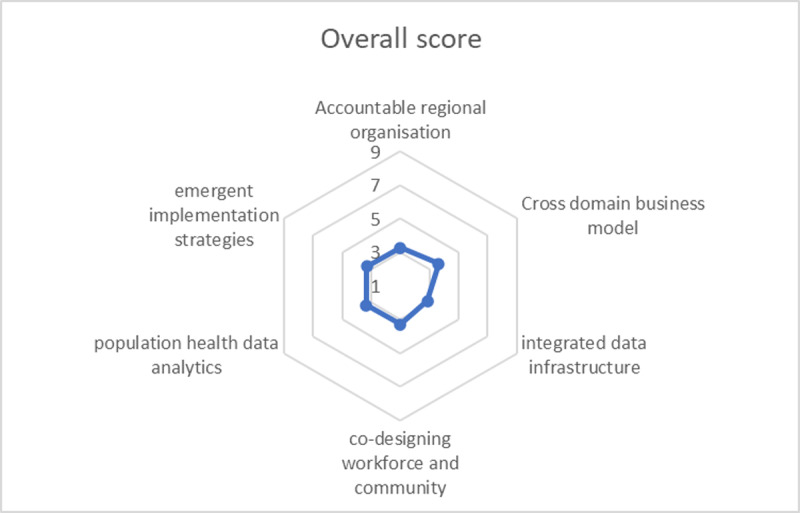
Average median scores per Population Health Management element of the PHM-MI.

Figure 3 below illustrates the overall results of the scores of the PHM-MI. It shows low median scores of three or lower (on a 9-point scale) across 69% (74/107) of all the items of the PHM-MI. This indicates a generally low-level of maturity for the Central Coast across the items that influence the successful implementation of Population Health Management. In Table 1, the scores are presented per PHM-element. Noteworthy are the low scores in the PHM-element ‘integrated data infrastructure’ with 84% (16/19) of the items scoring three or lower. Despite a significant availability of different data sets, this reflects the absence of elements necessary to facilitate the installation of an integrated data infrastructure on the Central Coast.

**Table 1:** Results of scoring the PHM-MI by Central Coast stakeholders.


	MEDIAN LOW (3 OR LOWER)	MEDIAN HIGH (7 OR HIGHER)	HIGH VARIABILITY (5 OR MORE)	HIGH AGREEMENT (ABOVE 0,7)

**Overall**	69% (74/107)	0%	40% (45/107)	45% (48/107)

**Accountable regional organisation**	75% (15/20)	0%	60% (12/20)	30% (6/20)

**Cross domain business model**	63% (12/19)	0%	42% (8/19)	63% (12/19)

**Integrated data infrastructure**	84% (16/19)	0%	32% (6/19)	68% (13/19)

**Codesign community and professionals**	57% (8/14)	0%	29% (4/14)	50% (7/14)

**Population health data analytics**	60% (9/15)	0%	33% (5/15)	40% (6/15)

**Emergent implementation strategies**	70% (14/20)	0%	50% (10/20)	20% (4/20)


### Qualitative results

#### Accountable regional organisation

Despite the existence of the Central Coast Health Alliance, and the development of the JSNA-process, the analysis demonstrates that there is no regional network or organisation working with a dedicated focus on improving the health and wellbeing of all community members. In the light of the recent pandemic, it was reported that close collaboration has been possible in the past across multiple stakeholders. While these networks around disasters – such as Covid, flooding and bush fires – have worked well, they were seen to be reactive to urgent needs and it was observed how such collaborations have not been ongoing beyond the disaster response period.

Participants also outlined many other ongoing issues hindering collaboration including: an ‘existing culture of keeping information internal’, ‘having an organizational inward focus’, and a ‘tendency to micro-management’ with reporting centred to each individual organization rather than across organizations. Another perceived barrier to collaboration has been the significant influence of political objectives on determining the separate strategies of the organizations involved, so determining their core operational and strategic focus. Therefore, long-term commitment to any local partnership has needed to work within the constraints of these political and regulatory challenges rather than be enabled by them. All in all, participants felt that the timing seemed right to formalize collaborations and expand the connections to support care integration, specifically to come up with a regional health perspective and a shared vision supported by several organizations, underpinned by available resources.

#### Cross domain business model

Participants perceived significant inequality across the different funding systems, highlighting the competitiveness in the system for resources and how these resources are allocated. However, many participants also saw opportunities for funding that could be made available for joint activities on the Central Coast. The main challenge articulated was that ‘every organization is on its own’ and did not often know about funds provided for similar projects or targeted to the same population groups – thus manifest in ongoing duplication in care and services without addressing some of the key system gaps identified by ALICE and other initiatives.

At the same time, participants reflected how this silo-based approach is impacted by the working culture, for example, in how budgets get dispersed down to the region at different timings for different organizations purposes; are earmarked to specific programs; and are usually tightly controlled and accounted for. This financial and regulatory environment has made it difficult to pool or manipulate these budgets for collaborative initiatives. Participants suggested that next steps would be to map what is available regarding budgets that might be shared, and how these might be accessed. In the long-term, funding for placed-based projects like ALICE need to become more aligned with the regional network’s aim and ambitions.

#### Integrated data infrastructure

The analysis highlighted a willingness to share data but that barriers from data privacy, governance and regulation often hindered efforts on data integration. Also, participants highlighted how the use of different platforms and IT systems ‘doesn’t help in that respect’ leading to data that is often only used for a single program. There are ‘lots of different datasets’, but they are seemingly created separately with no clear overview of what is available and how they are being used. Participants agreed that sharing what type of data there is within organizations and/or sharing data in an aggregated form could be a first step forward. For example, in using it for population health analysis (as achieved during the JSNA process), to share information between professionals, and to capture the voices from the community would provide possibilities of testing data sharing.

#### Co-designing workforce and community

There is a perceived “governance gap” when it comes to engagement with the community. While participants recognised the focus on understanding the needs of the Central Coast population, often the understanding of what should be done has not been followed up. The lack of connection between top-down processes (Chief Executive (CE)-level to community member) and bottom-up collaborations (community members to CE-level) was highlighted as a problem leading to ‘internal structural disconnection’. In contrast, participants were very positive about the collective focus on the needs of the Central Coast community. To build trust with the community, it was suggested that an overall strategy for community involvement be developed, with a guiding set of principles that is used across the Central Coast stakeholders regarding community involvement to make it a consistent experience to all community members.

#### Population health data analytics

Participants agreed that skilled data professionals were available to support data analytics, but that they have not yet been commissioned to work on creating an integrated data infrastructure (or service) to support larger data analyses. For a single organization, it was reported that it often costs too much to invest in both data professionals and data infrastructure leaving no in-house capability. However, the future option to ‘buy-in’ these skilled professionals to support a joint process of ‘commissioning’ was highlighted, specifically to support population segmentation and risk stratification to target specific populations for different interventions.

#### Emergent implementation strategies

Participants confirmed that the siloed working style across and within the organizations provided a challenging environment. The working style was considered ‘bureaucratic’ such that working on long-term goals was considered difficult given the need to account for immediate performance metrics and indicators. This challenging environment for change has led to perceptions of the lack of a ‘regional ambition’ despite written intent within strategies and the skills and expertise available to support such change. Also, participants felt that the learnings and experiences of past collaborations could have been better captured to support this, for example from collaboration successes during disaster response.

## Discussion

### Reflection on strengths and weaknesses of the Central Coast to support place-based care

For a fragmented health care system like Australia, the low propensity of most of the scores to be able to collectively manage approaches to place-based care were predictable. However, what was clear through the workshops was the development of a strategic intent towards addressing population health needs and place-based approaches to care that was largely absent five years previously. As reflected in the maturity scores, significant work on the structures, processes and working cultures is required to support the collaborative work that is needed. Using the PHM-MI tool could be used to benchmark progress in this respect for the Central Coast, potentially in comparison with other regions. Using the PHM-MI tool as part of the JSNA process on the Central Coast has informed the future strategic plans of the Central Coast Health Alliance and its partners. For example, CCLHD’s Clinical Services Plan 2023–28 outlines how collaboration with other service providers and the community is seen as an imperative and includes active consideration for regional service planning, as well as a commitment to enhancing primary and community services in place-based initiatives such as ALICE [16].

One of the challenges for the Central Coast identified from using the PHM-MI tool is to promote the joint understanding of what is needed to develop the right ‘accountable regional organization’ for place-based initiatives like ALICE. What should ALICE’s governance and accountability look like? How can local ALICE communities be integrated into this in ways that promote the voice of the local community and enable contextually-specific solutions to meet local needs? As ALICE progresses into its pre-implementation phase, and with the intention to support co-design and co-creation of ALICE with local communities and across regional stakeholders beyond the Alliance, it would be interesting to undertake a further PHM-MI analysis in future years (and across the wider stakeholder group) to see whether the extent to which the essential elements of effective PHM are maturing or not.

### Reflections on the use of the PHM-MI tool

Overall, when judging the maturity of the Central Coast to take forward PHM, the quantitative scores emerging from the survey matched the qualitative stories from participants during the workshops. However, as PHM was a new concept for a lot of participants, setting the scene for each PHM-element during the workshop phase was considered important to establish joint understanding and meaning of the results. The use of terminology throughout the tool was often regarded as too healthcare focused and created significant variability in interpretation of the items. For example, the term ‘Quadruple Aim’ used in healthcare as a means to drive quality and value in care delivery was interpreted by some participants as the ‘Quadruple Bottom Line’ which is used in governmental accounting to evaluate performance across cultural, economic, environmental and social factors. [19] The use of language in the tool was regarded as a key influence on the ability of different stakeholders to interpret and score the items. The number of participants and the position they have in the health and care system was also recognised as deeply influencing the nature of the results. Given just 13 people participated, mainly managers working in governmental organizations, the results only reflect their perspective. Therefore, they can only be considered as a first exploration of the strengths and weaknesses of the Central Coast region for PHM.

This case analysis demonstrates that the PHM-MI can be an effective tool to gain insights into the strengths and weaknesses of the region towards PHM. For other initiatives, this case illustrates certain pressure points for using such a tool effectively. The three main points being: the selection of participants in the process; the conversation it brings being more important than the numerical scores; and how it should be embedded or backed by an ongoing strategic development towards sustainable collaboration. Existing literature on the use of tools like PHM-MI, such as the Project INTEGRATE Framework, present similar conclusions in how learning from them can best be derived [20].

On the Central Coast, the process of applying the PHM-MI was a means to building relationships. It brought stakeholders together to discuss each other’s perspective and how to interpret the items for their context. While decisions were made in other stakeholder settings, the workshops and the final case report supported the lobby for the development and implementation of ALICE. Ongoing discussions about appropriate governance arrangements for ALICE have since supported the creation of an Executive Steering Group of the Alliance now extended to the local council and Department of Regional NSW. Meanwhile, local residents’ committees and provider collaboratives are planned to ensure ongoing mechanisms for neighbourhood consultation and co-creation.

### Prospects for the future use of the PHM-MI tool

This case study project has demonstrated that the PHM-MI can be used to inform change when embedded within ongoing strategic processes in a region seeking to improve population health. While the tool was developed in the Netherlands, stakeholders on the Central Coast recognized the items and enabled a shared understanding of current strengths and weaknesses and priority areas for improvement. As the uptake of PHM grows, tools such as the PHM-MI are important to support stakeholders and communities to prioritise local health needs. As the tool was developed in a European setting and now tested in Australia, this confirms the potential for the adaptation of the tool for use in other contexts and settings internationally. With application in other health systems, the trade-off for future development will be between adapting the tool to be a perfect fit for the local context and the ability to use the tool to compare PHM implementation across different settings.

In terms of future application, the development of the PHM-MI tool on the Central Coast has not gone unnoticed. Together with a consultancy company, the tool has undergone a next iteration of development for use in Australia and internationally where it has been fitted into a digital data collection and analytical platform. Having this platform will potentially allow the development of an international database to compare regions across health systems and contexts and form guidance on the best way to support PHM. In turn, this knowledge can inform practice on how to best implement PHM to improve the health and wellbeing of their population in a sustainable way. In Australia, the tool is being developed to help Primary Health Networks and their partners understand their direct influence and their strategic influence, so supporting their commissioning function.

### Lessons learned

- Using the PHM-MI to map the strengths and weaknesses of the Central Coast to enable place-based care has started a conversation about prerequisites and key issues to support future initiatives such as ALICE.
- Bringing regional stakeholders together to use the PHM-MI tool was challenging but was enabled by it being embedded within a pre-existing regional strategic process to examine population health needs.
- The PHM-MI can be translated for use in any regional or national context, potentially supporting benchmarking opportunities.
- Using the PHM-MI tool effectively requires the creation of time and space amongst stakeholders to reflect upon the results to inform future action.

## Conclusion

Overall, the Central Coast currently exhibits low maturity to support the implementation of PHM, an observation that is likely to be true across much of NSW and Australia. The most significant barrier appears to be the ‘inward focus’ of organizations that is reflected in the scores provided to almost all the PHM-elements. However, using the PHM-MI tool to map the capability of the Central Coast has started new conversations about future prerequisites that could support collaborative place-based initiatives. It has also demonstrated the potential of the tool to be used in different regions across the world. Scaling up the use of the tool has the potential to create an international database for comparative research, for example to analyse what the most successful items are when working towards a PHM approach. In turn, this knowledge can feed back into practice to inform and guide regions on how to best implement PHM sustainably.

## References

[1] Stein KV, Miller R, Aldasoro E, Goodwin N. Always Look on the Bright Side – Lessons Learned from Another Decade of Integrating Care. International Journal of Integrated Care; 2022. DOI: 10.5334/ijic.7513 PMC9716999 36483482

[2] van Ede AFTM, Minderhout RN, Stein KV, Bruijnzeels MA. How to successfully implement population health management: a scoping review. BMC Health Services Research. 2023; 23(1): 910. DOI: 10.1186/s12913-023-09915-5 37626327 PMC10464069

[3] Alderwick H, Ham C, Buck D. Population health systems: going beyond integrated care. Kings Fund; 2015.

[4] Swarthout M, Bishop MA. Population health management: Review of concepts and definitions. American Journal of Health-System Pharmacy. 2017; 74(18): 1405–11. DOI: 10.2146/ajhp170025 28887342

[5] Bardsley M, Steventon A, Smith J, Dixon J. Evaluating integrated and community-based care: how do we know what works? Nuffield Trust; 2013. https://citeseerx.ist.psu.edu/.document?repid=rep1&type=pdf&doi=cbc18797bbb5716784a4c8b76a6c19428ab45fcd.

[6] Eastwood J, Barmaky S, Hansen S, Miller E, Ratcliff S, Fotheringham P, et al. Refining Program Theory for a Place-Based Integrated Care Initiative in Sydney, Australia. Int J Integr Care; 2020. DOI: 10.5334/ijic.5422 PMC7528678 33041730

[7] Goodwin N. Understanding Integrated Care. Int J Integr Care. 2016; 16(4): 6. DOI: 10.5334/ijic.2530 PMC5354214 28316546

[8] WHO. Population health management in primary health care: a proactive approach to improve health and well-being. Copenhagen: WHO Regional Office for Europe; 2023. https://www.who.int/europe/publications/i/item/WHO-EURO-2023-7497-47264-69316.

[9] van Ede AFTM, Stein KV, Bruijnzeels MA. Assembling a population health management maturity index using a Delphi method. BMC Health Services Research. 2024; 24(1): 110. DOI: 10.1186/s12913-024-10572-5 38243278 PMC10799527

[10] Kolukısa Tarhan A, Garousi V, Turetken O, Söylemez M, Garossi S. Maturity assessment and maturity models in health care: A multivocal literature review. DIGITAL HEALTH. 2020; 6: 2055207620914772. DOI: 10.1177/2055207620914772 32426151 PMC7216018

[11] Pavlickova A, Henderson D, Alexandru C, Alhambra T. The maturity of integrated care systems: lessons learned in using the SCIROCCO tool across Europe. European Journal of Public Health. 2018; 28(suppl_4). DOI: 10.1093/eurpub/cky213.052

[12] CCLHD. Central Coast Local Health District – about us. Available from: https://www.cclhd.health.nsw.gov.au/about-us/.

[13] HNECCPHN. Hunter New England Central Coast Primary Health Network. Available from: https://thephn.com.au/about-us.

[14] Australian Government. The Australian Health and Care System. In infographic Ashl, editor. *Department of Health and Aged Care*. https://www.health.gov.au/resources/publications/australias-health-landscape-infographic.

[15] CCLHD. Caring for the coast strategy 2019–2024. https://www.cclhd.health.nsw.gov.au/wp-content/uploads/Strategic_Plan_2019-2024.pdf.

[16] CCLHD. Clinical Services Plan 2023–2028. Central Coast Local Health District; 2023. https://www.cclhd.health.nsw.gov.au/wp-content/uploads/CCLHD_Clinical_Services_Plan_2023-2028.pdf.

[17] Harris PA, Taylor R, Minor BL, Elliott V, Fernandez M, O’Neal L, et al. The REDCap consortium: Building an international community of software platform partners. Journal of Biomedical Informatics. 2019; 95: 103208. DOI: 10.1016/j.jbi.2019.103208 31078660 PMC7254481

[18] Harris PA, Taylor R, Thielke R, Payne J, Gonzalez N, Conde JG. Research electronic data capture (REDCap)—A metadata-driven methodology and workflow process for providing translational research informatics support. Journal of Biomedical Informatics. 2009; 42(2): 377–81. DOI: 10.1016/j.jbi.2008.08.010 18929686 PMC2700030

[19] Bodenheimer T, Sinsky C. From triple to quadruple aim: care of the patient requires care of the provider. Ann Fam Med. 2014; 12(6): 573–6. DOI: 10.1370/afm.1713 25384822 PMC4226781

[20] Calciolari S, González Ortiz L, Goodwin N, Stein V. Validation of a conceptual framework aimed to standardize and compare care integration initiatives: the project INTEGRATE framework. Journal of Interprofessional Care. 2022; 36(1): 152–60. DOI: 10.1080/13561820.2020.1864307 33761800

